# The British Medical Association and Medical Defence

**Published:** 1896-07-25

**Authors:** 


### THE BRITISH MEDICAL ASSOCIATION AND MEDICAL DEFENCE.

The decision arrived at by the general meeting of the British Medical Association recently held at Birmingham, to apply for an alteration of their memorandum of association so that they may be able to undertake the work of "medical defence" in addition to their other duties, is of interest not only to members of the Association but to the rest of the profession. To the members the new move partakes of the nature of a large commercial speculation, on the success or non-success of which may hinge the future prosperity of the Association, and we are not surprised to find the treasurer entering a protest against so serious a step being taken without a definite scheme of medical defence being first submitted to the branches. To the unattached members of the profession the scheme also has a somewhat serious aspect, for it is impossible to remain oblivious to the fact that, according to the form the scheme may take, it may depend whether or not existing agencies for medical defence, which at present are open to the whole profession, will be able to hold their own in face of the opposition of so widely ramifying a body as the British Medical Association. Mr. Butlin and Professor Victor Horsley were then in perfect order in asking for the scheme to be produced and discussed before such alterations are made in the constitution of the Association. The Chairman was, no doubt, technically in the right in saying that the meeting had nothing to do with the particular scheme which was ultimately to be adopted; but the fact remains, as Mr. Butlin pointed out, that at least four different views are held by members on the subject, and that the question what scheme is to be adopted is the very essence of the question whether such powers as are asked for shall be given to the Council.

According to the resolution passed at Birmingham, everything is thrown upon the Council. Every clause dealing with medical defence contains the phrase, "in such manner as the Council of the Association may think fit," or one to the same effect, and not a word is said about any referendum to the branches; and, although the branches are, no doubt, represented on the Council, it is worthy of the most careful consideration by the whole mass of members of the Association whether this body is so directly representative as to make it desirable to hand over to them such very large powers as are asked for. Especially is this caution necessary from the fact that, while they are asking for this great display of confidence in them, the Council are themselves propounding a scheme which is so far from being generally acceptable that, even from within their own body it is already traversed by two rival plans which differ radically both from the official scheme and from each other. It is not for us to enter into any criticism of these rival proposals, but we may fairly urge that in view of such lack of unanimity the members of the Association, before granting such large powers, may fairly ask what will they do with them?

In regard to this it is not without interest to note that while the new powers asked for have relation to the "defence, promotion, or maintenance of the honour or interests of the medical profession," or the doing such things as may be "directly or indirectly conducive to the interests of the medical profession," and while not a word is said in the proposed alteration of the memorandum of association as to the interests of its own members being dealt with separately from those of the medical profession at large, the very first thing we notice in the scheme propounded by the Council is "that only members of the Association who pay the voluntary subscription to the Medical Defence Fund should be entitled to claim individual assistance from that fund." Of course, if once the alterations received the sanction of the Board of Trade and the High Court the Council will have power to act as they "may think fit"; but it is hardly to be expected that the Court will allow such an alteration to be made when the very body to whom the powers asked for are to be entrusted have already put forth a scheme according to which they propose to exercise these powers, not alone, as stated in the memorandum, for the defence of "the medical profession," but especially for the defence of a select few of its own members.

The meeting at Carlisle seems likely, then, to be an important one in the annals of the Association. If the resolution of the Council should be passed it might turn out to be the commencement of a movement which, while of the greatest utility to the members of the Association, might do considerable injury to those who are not within its ranks. At the same time it must not be forgotten that if the proposed scheme of medical defence should ever get to work it might involve the Association in considerable expense while tampering with the very foundations of its financial success, for if the subscription to the defence fund were made obligatory, what is to many the main attractiveness of membership would be removed, viz., the supply of a journal at a comparatively low price. After all, there are objections to killing the goose that lays the golden eggs.

